# Upregulation of the miR-17-92 cluster and its two paraloga in osteosarcoma – reasons and consequences

**DOI:** 10.18632/genesandcancer.6

**Published:** 2014-01

**Authors:** Leila Arabi, Joël R Gsponer, Jan Smida, Michaela Nathrath, Valeria Perrina, Gernot Jundt, Christian Ruiz, Luca Quagliata, Daniel Baumhoer

**Affiliations:** ^1^ Institute of Pathology, University Hospital Basel, Basel, Switzerland; ^2^ Nanotechnology Research Center, School of Pharmacy, Mashhad University of Medical Sciences, Mashhad, Iran; ^3^ Clinical Cooperation Group Osteosarcoma, Helmholtz Zentrum Muenchen, German Research Center for Environmental Health, Neuherberg, Germany; ^4^ Bone Tumor Reference Center at the Institute of Pathology, University Hospital Basel, Basel, Switzerland; ^5^ Shared senior authorship

**Keywords:** osteosarcoma, miR-17-92, miR-106a-363, miR-106b-25, FAS, BIM

## Abstract

Osteosarcomas (OS) are aggressive bone tumors characterized by complex karyotypes with highly variable structural and numerical chromosomal aberrations. Although several genes and pathways commonly altered in malignant tumors have also been identified in OS, the molecular pathogenesis and driving genetic events eventually leading to tumor development are still poorly understood. The microRNA (miRNA) cluster 17-92 and its two paraloga 106a-363 and 106b-25 are known to have diverse oncogenic properties and have been shown to be constantly upregulated in several established OS cell lines. In this study we analyzed a series of 75 well characterized pretherapeutic OS samples for their expression of cluster-related miRNAs and correlated our findings with clinico-pathological parameters including prognosis, metastases and response to neoadjuvant therapy. Interestingly, higher expression levels of specific miRNAs were significantly associated with an adverse outcome of patients and were also higher in patients with systemic spread. We could furthermore show a direct correlation between the expression of cluster activators (MYC, E2F1-3), inhibitors (TP53), individual miRNAs, and pro-apoptotic targets (FAS, BIM). Our findings therefore underline a critical role of the miR-17-92 cluster and its two paraloga in OS biology with pathogenetic and prognostic impact.

## INTRODUCTION

Osteosarcomas (OS) are the most common primary malignant tumors of bone generally affecting the metaphyses of long bones in children and adolescents [1]. Due to a high rate of systemic spread already at the time of diagnosis patients greatly benefit from (neo-) adjuvant polychemotherapy in addition to radical surgery and reach 10-year survival rates of up to 73% in case of good response to cytostatic regimens [2, 3]. However, a substantial group of patients with metastatic, recurrent and/or refractory disease still lacks effective treatment options underlining the urgent need for new therapeutic alternatives and targets. Furthermore, there are no established biomarkers in OS that could identify patients with particularly aggressive tumors and could therefore constitute a basis for a more individualized treatment stratification [4]. One reason for this phenomenon is the genetic heterogeneity and complexity that is characteristic for OS and which hampers the identification of initiating and/or sustaining oncogenetic drivers. Amongst the most commonly mutated and/or altered genes in OS, TP53 and MYC have been identified, both of which are known to be deregulated in a variety of malignant tumors [1, 4].

**Figure 1:**
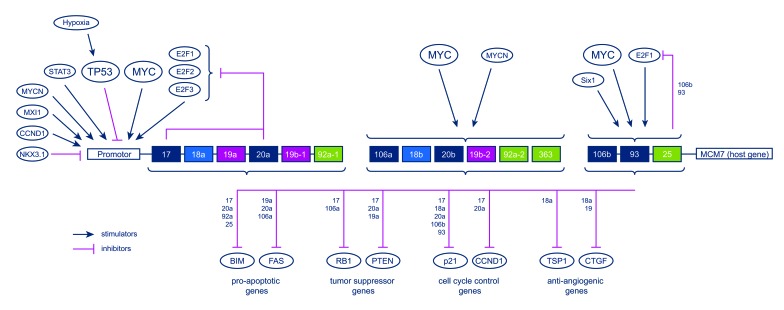
The miR-17-92 cluster and its two paraloga miR-106a-363 and miR-106b-25 are centered in a complex network of regulators (upper half) and targets (lower half) of which this scheme only shows a selection for a better overview [8, 9].

Besides conventional oncogenes and tumor suppressors, microRNAs (miRNA) have increasingly been recognized as regulators of gene expression that can acquire oncogenic potential. The miR-17-92 cluster, also named oncomir-I, and its two paraloga miR-106a-363 and miR-106b-25 were among the first families of those small RNA molecules that were found to be upregulated in several malignant tumors. Meanwhile, several cluster-related miRNAs were shown to accelerate tumor development, to induce angiogenesis, to prevent apoptosis, and, only recently, to crucially influence osteoblastic proliferation and differentiation [5-7]. All three clusters are part of elaborate regulatory networks and can influence the expression of various genes involved in cell cycle control, apoptosis and angiogenesis (Figure 1). Interestingly, MYC is known to stimulate the expression of cluster-related miRNAs whereas TP53 seems to have an inhibitory effect [8, 9]. In a previous study we demonstrated the upregulation of several of the respective miRNAs in a panel of established OS cell lines (HOS58, U2-OS, Saos-2, MNNG/HOS, SJSA-1, and MG-63) which was meanwhile confirmed by an independent group [10, 11].

In this study, we assembled a series of well characterized pretherapeutic OS samples to validate our cell line results in tumor biopsies and to analyze if the expression of individual miRNAs correlated with clinico-pathological parameters including prognosis, metastatic disease and/or response to therapy. In a next step, we interrogated the expression of selected regulators (MYC, TP53, E2F1, E2F2, E2F3) and pro-apoptotic targets (FAS, BIM) of the miR-17-92 cluster and its two paraloga that have already been described to show an altered expression and potential pathogenetic impact in OS [1, 4, 12, 13]. By this means, we aimed to confirm the upregulation of cluster related miRNAs and to provide a deeper insight into the causes and consequences of cluster activation in human osteosarcoma.

## RESULTS

### Patients´ and samples´ characteristics

All patients´ characteristics are presented in Table 1. MicroRNA stability is known to efficiently enable expression analyses in a variety of tissue sources. In this study we extracted miRNAs from 75 formalin fixed paraffin embedded (FFPE) pretherapeutic osteosarcoma samples. The expression levels of all miRNAs were evaluable in 57/75 cases and of selected regulators and targets of the respective miRNA clusters in 41/75 cases (Table 1). Causes of samples exclusion were either insufficient amounts of tissue or poor RNA quality.

**Table 1:** Patients´ characteristics

		Complete series (n=75)	miRNA series (n=57)	Regulator / target series (n=41)
Gender		75/75 (100%)	57/57 (100%)	41/41 (100%)
	male	38	29	21
	female	37	28	20

Age at diagnosis (years)		75/75 (100%)	57/57 (100%)	41/41 (100%)
	average	23.5	24.9	23.8
	median	16	16	16
	range	7-82	7-82	7-79

Metastastatic spread		75/75 (100%)	57/57 (100%)	41/41 (100%)
	yes	22	15	11
	no	53	42	30

Observation period (months)		75/75 (100%)	57/57 (100%)	41/41 (100%)
	average	34.6	27.4	35.2
	median	18	15	20
	range	0-142	0-112	0-117

Response to treatment		49/75 (65%)	37/57 (64.9%)	26/41 (63.4%)
	good	25	19	12
	poor	24	18	14

Recurrence		75/75 (100%)	57/57 (100%)	41/41 (100%)
	yes	15	11	7
	no	60	46	34

Survival		75/75 (100%)	57/57 (100%)	41/41 (100%)
	alive	62	46	34
	deceased	13	11	7

Location		75/75 (100%)	57/57 (100%)	41/41 (100%)
	Femur	32	23	18
	Tibia	17	12	8
	Jaws	7	7	5
	Humerus	5	5	3
	Fibula	5	2	2
	Pelvis	4	4	3
	Spine	4	4	2
	Ulna	1	-	-

### MicroRNA expression in osteosarcoma

To date no comprehensive evaluation of the miR-17-92 cluster and its two paraloga has been carried out in OS. Our work revealed that several cluster-related miRNAs were constantly upregulated in the tumors analyzed compared to both human osteoblasts and mesenchymal stem cells (Figure 3a). Importantly, higher expression levels of various miRNAs were significantly associated with an adverse overall survival: miR-19a (p = 0.037), miR-20a (p = 0.024), miR-19b (p = 0.031), miR-92a (p = 0.006), and miR-106b (p = 0.032) (Figure 3b and 3c). A similar trend could be shown also for all the residual miRNAs but it did not reach statistical significance (data not shown). We further investigated a potential correlation of the miRNAs with metastatic spread and with response to neoadjuvant chemotherapy. For all miRNAs analyzed, metastatic OS showed higher expression levels of the respective miRNAs than tumors without systemic spread (Figure 3d). However, statistical significance was not reached. There was no correlation between higher expression levels of cluster-related miRNAs and a poor response to chemotherapy (data not shown).

**Figure 2:**
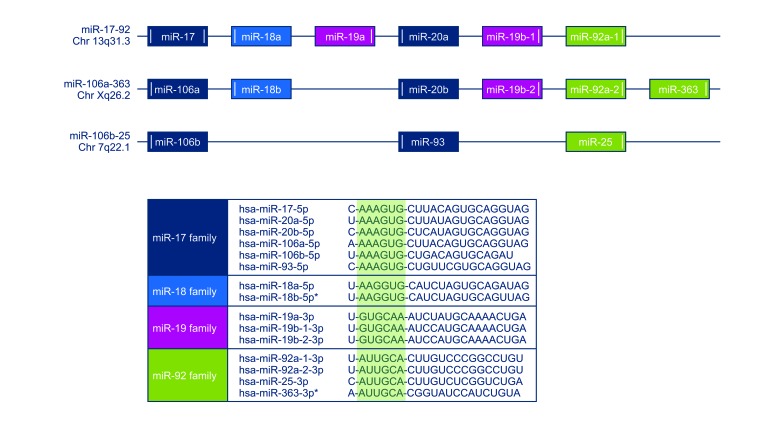
The miRNA clusters are transcribed in polycistronic transcripts and organized in four families with high sequence homology (* = not included in the analysis).

**Figure 3:**
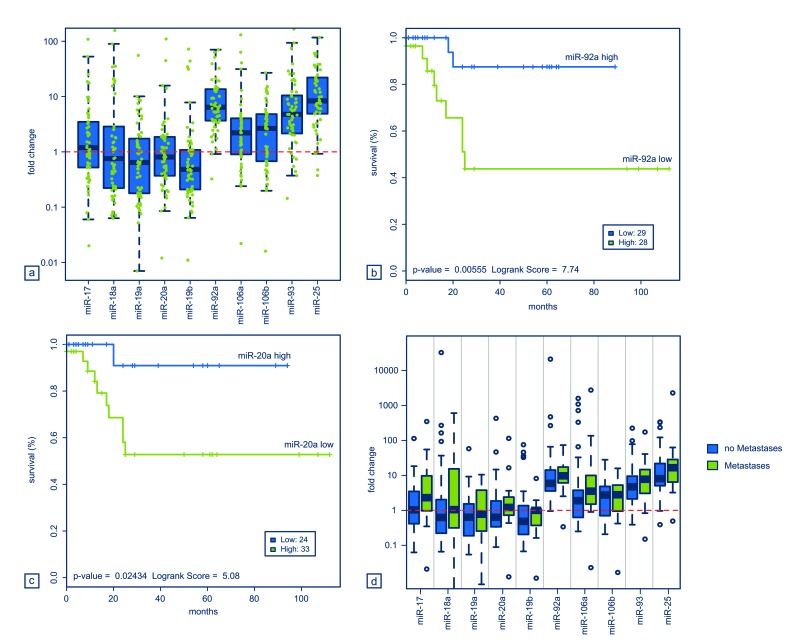
Relative miRNA expression of all analyzed tumors compared to the corresponding expression in L87/4 cells (a). Kaplan-Meier plots showing an adverse outcome for OS patients with higher expression levels miRNAs miR-92a (b) and miR-20a (c). Statistical significance was assessed by means of the logrank test. Relative miRNA expression of all analyzed tumor stratified by metastatic spread (d).

### Expression of selected regulators

**Figure 4:**
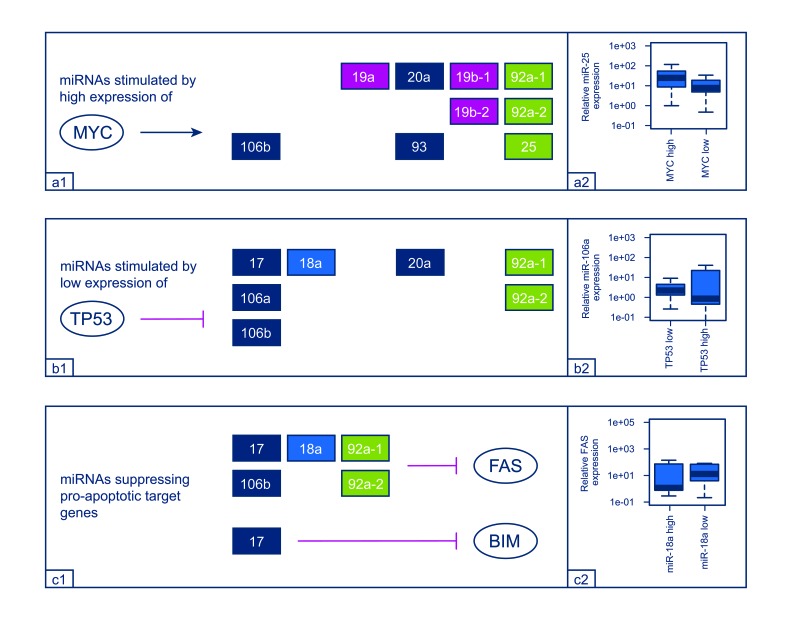
Comparison between regulator / target gene and miRNA expression levels. Higher expression levels of MYC showed a trend towards concomitant higher expression levels of the depicted miRNAs (a1) which is presented exemplarily for miR-25 in a separate box-plot (a2). Representing a cluster inhibitor, lower expression levels of TP53 correlated with higher expression levels of the presented miRNAs (b1). Again, miR-106a is shown exemplarily in a separate box plot (b2). Higher expression levels of the miRNA depicted revealed a trend towards concomitant lower expression levels of the pro-apoptotic targets FAS and BIM (c1). Exemplarily, the association between FAS and miR-18a expression is shown in a separate box plot (c2).

MicroRNAs are generally centered in complex networks of regulatory genes complicating the possibility to identify direct partner interactions. Taking advantage of previous work, we have selected a number of potential direct regulators of the miR-17-92 cluster and its two paraloga and analyzed their expression in our cohort of osteosarcoma samples. As expected, higher expression levels of MYC, E2F1, E2F2, and E2F3 showed a trend towards concomitant higher expression levels of most of the analyzed miRNAs, in line with the concept of those genes to represent stimulators of the respective clusters (Figure 4a). For TP53 as an inhibitor of the cluster-related miRNAs, the opposite effect was observed in the vast majority of cases (Figure 4b). However, statistical significance was only detected for E2F1 / E2F2 and miR-25 as well as for E2F3 and miR-20a / miR-93 (data not shown).

### Expression of selected Targets

Driven by sequence specific targeting, miRNAs have been shown to influence a plethora of different genes. Here we exemplarily tested whether the expression levels of cluster-related miRNAs could be correlated with predicted and pro-apoptotic target genes that are known to be frequently downregulated in osteosarcoma. Indeed, higher expression levels of some miRNAs (miR-17, miR-18a, miR-92a, and miR-106b) showed a trend towards downregulation of FAS. For BIM, only miR-17 revealed a similar effect (Figure 4c). None of those inversed relations reached statistical significance.

## DISCUSSION

In the series of pretherapeutic OS samples analyzed in the present study, several members the miR-17-92 cluster and its two paraloga were constantly upregulated in the vast majority of tumors and the expression levels of individual miRNAs correlated with an adverse outcome of patients and metastatic spread. Amplification or elevated expression of the cluster-related miRNAs has previously been shown in various other malignant tumors and has also been linked to an unfavourable prognosis of patients [8, 20]. However, this is the first study revealing a prognostic impact of these miRNA clusters in human OS. Since metastatic spread is the most adverse prognostic factor in OS, elevated miRNA expression associated with both prognosis and metastases is not surprising [1]. The response to neoadjuvant chemotherapy was nonetheless not correlated to miRNA expression levels which might be influenced by the relatively low number of cases for which this information was available (Table 1).

The regulators stimulating and suppressing the cluster-related miRNA as well as the targets of the individual miRNAs form an elaborate network of genes and pathways commonly deregulated in cancer (Fig. 1) [8, 9]. Since many functional interdependencies and feedback loops are involved, the identification of direct relations and interactions between isolated network members is difficult to accomplish. For this study, we chose known stimulators (MYC, E2F1-3) and inhibitors (TP53) of the miRNA clusters that are frequently amplified, elevated or inactivated in OS and could show that all of those regulators correlated with an up- or downregulation of the corresponding miRNAs in most of the cases as anticipated. Moreover, MYC amplification has been shown to represent an adverse prognostic factor in OS as our results also point out for several miRNAs stimulated by MYC [21]. TP53 is affected by germ line mutations, LOH or deletions in approximately 40% of OS and although the expression level of TP53 can certainly not reflect the complete functional status of the gene, tumors with low expression of TP53 showed relatively higher levels of the respective miRNAs. Importantly, the comparison of expression levels can only indicate but not definitely proof an interplay between regulators and miRNAs. On the other hand, some of the most commonly altered genes in OS all seem to participate in stimulating the expression of the miR-17-92 cluster and its two paraloga providing a rationale for further investigation.

In a recent work by Huang and colleagues, higher expression levels of miR-20a were shown to suppress FAS in several OS cell lines and were associated with higher rates of metastatic spread in a mouse model [13]. Similarly, in gastric cancer, inhibition of FAS-mediated apoptosis was linked to an overexpression of the cluster-related miR-106a which was shown in cell lines but also in FFPE samples [22]. In our series, we found that higher expression levels of miR-17, miR-18a, miR-92a, and miR-106b correlated with lower levels of FAS. Since the respective miRNA clusters are transcribed in polycistronic transcripts and organized in four different families with high sequence homology, our results might indicate a certain amount of interchangeability of the cluster-related miRNAs as miR-20a and miR-106a are part of the same miRNA-family like miR-17 and miR-106b (Fig. 2). BIM represents another gene with pro-apoptotic properties that is suppressed by the miR-17-92 cluster in various tumors but also in osteoblasts [23]. In our series, only miR-17 was correlated with a downregulated BIM expression.

Understanding the molecular pathogenesis of OS including the reasons for tumor development, mechanisms of systemic spread and primary chemoresistance in a subgroup of patients is the prerequisite for a more individualized treatment stratification and for identifying urgently needed new and innovative therapeutic targets. The highly variable genetic alterations found in OS which at least in a subgroup of tumors results from chromothripsis with random chromosomal rearrangements, makes driving genetic events difficult to determine [24, 25]. Several genes and pathways involved and altered in OS, however, seem to be part of the regulatory network the miR-17-92 cluster and its two paraloga miR-106a-363 and miR-106b-25 are centred in [1, 4, 8, 9]. RB1 for example is commonly mutated in OS but miRNA-mediated silencing of the gene might be an alternative mechanism to inactivate the tumor suppressor properties of RB1 [26, 27]. The constant upregulation of the cluster-related miRNAs as shown here might therefore indicate a superordinate role of the clusters in OS biology and a point of intersection at which several oncogenetic pathways and events converge. Further functional studies are needed to prove if an upregulation of cluster-related miRNAs is indeed mandatory for OS development and progression and therefore might represent a new therapeutic target as was proposed recently [28]. The compelling prognostic impact of elevated miRNA expression shown here, however, clearly points to a central pathogenetic role of the three miRNA clusters in OS biology.

## MATERIAL & METHODS

### Tumor samples and cell lines

75 cases of pretherapeutic OS samples with detailed clinico-pathological data were selected from the archives of the Bone Tumor Reference Center in Basel. Clinico-pathological data included follow-up, rates of metastatic spread and recurrent disease as well as response to neoadjuvant treatment.

Since there is no normal or adjacent tissue that OS are derived from and most likely mesenchymal stem cells with at least some extent of osteoblastic differentiation represent the source of tumor development, cultured human osteoblasts (hFOB 19.9) and mesenchymal stem cells (L87/4) were used as normal controls. Cells were grown and subsequently transferred into paraffin blocks according to routine protocols to ensure similar conditions for further workup as for the tumor samples.

### MicroRNA extraction

MicroRNAs extraction was performed using the High Pure miRNA Isolation Kit (Roche, Switzerland) according to the manufacturer´s instructions. RNA purity and quantity were measured using the 2100 Bioanalyzer (Agilent Technologies, Germany).

### Quantitative Real-time Reverse-transcription polymerase chain reaction

The expression levels of various miRNAs included in the miR-17-92 cluster and its two paraloga miR-106a-363 and miR-106b-25 (Figure 2) were determined using miRCURY™ LNA Universal RT microRNA PCR analysis (Exiqon, Denmark). The two-step protocol involved universal reverse transcription (RT) to convert miRNAs into complementary DNA, followed by real-time quantitative PCR amplification using microRNA-specific and LNA™-enhanced forward and reverse primers. The small non-coding RNA SNORD44 was used as an endogenous control and NEW ExiLENT SYBR® Green was applied for PCR product detection and quantification (Exiqon, Denmark). All samples were analyzed in triplicates. To calculate the relative expression levels of all miRNAs, the 2^−ΔΔCt^ cycle threshold method was applied using SNORD44 and hFOB 19.9 or L87/4 for normalization purposes [14].

For validation of selected regulators and targets of the analysed miRNAs, the relative expression levels of TP53 (probe Hs01034249_m1), MYC (probe Hs00153408_m1), E2F1 (probe Hs00153451_m1), E2F2 (probe Hs00231667_m1), E2F3 (probe Hs00605457_m1), FAS (probe Hs00236330_m1), and BIM (probe Hs00708019_s1) were determined by using the TaqMan® Probe-Based Gene Expression Analysis (Applied Biosystems, Life Technologies, Switzerland). The 18S housekeeping probe and hFOB 19.9 or L87/4 cells were used for normalization. All primers were purchased from Applied Biosystems (Life Technologies, Switzerland).

### Statistical Analysis

In order to assess the association between the different relative miRNA expression levels and evaluate their clinical significance we conducted an exploratory statistical analysis. Statistical differences between groups were calculated using a Wilcox-rank sum test and specific cut-offs for grouping the variables into “low” and “high” groups were calculated by maximizing the log rank score in a time dependent survival analysis. The significance level was set to 0.05. The statistical analysis was done using R Version 3.0.1 [15], including different packages: “MASS” [16]; “coin” [17]; “survival” [18, 19].

### Funding

Daniel Baumhoer and Gernot Jundt were supported by the Forschungsfonds of the University of Basel (DB) and by the Foundation for the Preservation of the Basel Bone Tumor Reference Center (DB and GJ). Jan Smida and Michaela Nathrath belong to the Translational Sarcoma Research Network supported by the BMBF (FKZ01GM0870).

### Ethical approval

The study was approved by the local ethical committee of Basel, Switzerland (EKBB, Reference 274/12).
